# Human Microphysiological Models of Intestinal Tissue and Gut Microbiome

**DOI:** 10.3389/fbioe.2020.00725

**Published:** 2020-07-31

**Authors:** Steven N. Steinway, Jad Saleh, Bon-Kyoung Koo, Delphine Delacour, Deok-Ho Kim

**Affiliations:** ^1^Department of Medicine, Johns Hopkins University School of Medicine, Baltimore, MD, United States; ^2^Cell Adhesion and Mechanics, Institut Jacques Monod, CNRS UMR 7592, Paris Diderot University, Paris, France; ^3^Institute of Molecular Biotechnology, Austrian Academy of Sciences (IMBA), Vienna Biocenter (VBC), Vienna, Austria; ^4^Department of Biomedical Engineering, Johns Hopkins University School of Medicine, Baltimore, MD, United States

**Keywords:** microphysiological model, gut-on-a-chip, organ chip, microbiome, intestinal tissue, organoid, microfluidics

## Abstract

The gastrointestinal (GI) tract is a complex system responsible for nutrient absorption, digestion, secretion, and elimination of waste products that also hosts immune surveillance, the intestinal microbiome, and interfaces with the nervous system. Traditional *in vitro* systems cannot harness the architectural and functional complexity of the GI tract. Recent advances in organoid engineering, microfluidic organs-on-a-chip technology, and microfabrication allows us to create better *in vitro* models of human organs/tissues. These micro-physiological systems could integrate the numerous cell types involved in GI development and physiology, including intestinal epithelium, endothelium (vascular), nerve cells, immune cells, and their interplay/cooperativity with the microbiome. In this review, we report recent progress in developing micro-physiological models of the GI systems. We also discuss how these models could be used to study normal intestinal physiology such as nutrient absorption, digestion, and secretion as well as GI infection, inflammation, cancer, and metabolism.

## Introduction

The human gastrointestinal (GI) tract is the site of ingestion and digestion of nutrients, nutrient absorption, secretory function, and elimination of waste product (Trowers and Tischler, 2014). The GI tract is a tubular structure which is composed of three main compartments: a muscular layer surrounding a mucous membrane and a lumen.

The GI tract is divided into four layers: the mucosa (epithelium, lamina propria, and muscular mucosae), the submucosa, the muscularis propria (inner circular muscle layer, intermuscular space, and outer longitudinal muscle layer), and the serosa (Jaladanki and Wang, 2011). An intrinsic nervous system called the enteric nervous system (ENS) helps regulate the muscular compartment and epithelial cells. The ENS is a dense network of neurons present throughout the GI. However, its composition, neuronal density and morphology varies according to the digestive segment. Together with the muscular layers, it regulates intestinal motility, peristalsis, which is responsible for migration of the food bolus along the digestive tract. Moreover, they provide a mechanical basis for the establishment of the mucosal architecture *per se* during development. In fact, Shyer et al. (2013) showed that, in addition to the endodermal signaling, smooth muscle differentiation is required for intestinal tissue shaping and villus formation (Walton et al., 2016). Further, there is growing evidence that gut microbiota contribute to gut motility (Quigley, 2011). Moreover, the ENS is part of the “gut-brain axis” and, because of its autonomous property, is nowadays considered a “second brain” (Cryan and Dinan, 2012; Mayer et al., 2015; Martin et al., 2018).

**FIGURE 1:**
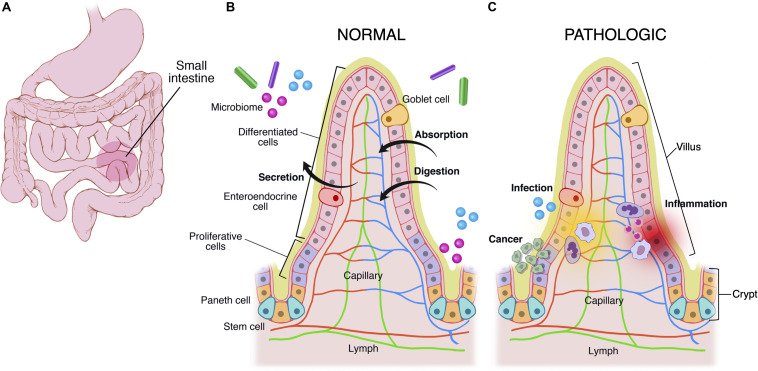
Multiscale physiological features of small intestine of the gastrointestinal tract. The GI tract **(A)** grossly consists of the esophagus, stomach, small intestine, large intestine, and rectum. The small intestine is made of crypts and villi **(B,C)**. Crypts are home to the LGR5+ intestinal stem cell, which differentiates into paneth cells, enteroendocrine, goblet cells, and enterocytes. As cells differentiate they migrate up the villus. These cells are covered in a mucus layer and the microbiome, which are all involved in normal homeostatic functions of immune regulation, secretion, absorption, and digestion. **(C)** Infection, cancer, and inflammatory processes disrupt the gastrointestinal homeostasis.

The mucous membrane is composed of a muscularis mucosae, a columnar epithelial monolayer and a mucus gel. This general structure is maintained throughout the GI tract, and most structural variations occur in the mucosal layer. For example, the stomach is made of a secretory mucosa for digestion, whereas crypt-villus subunits exist in the small intestine for absorption and secretion (Figure 1). In comparison to other epithelia, the epithelial monolayer lining the small intestine has a simple and regular architecture, where proliferative and differentiated cells are distributed in distinct areas (Figure 1B). It is characterized by its organization into finger-like tissue shapes protruding into the intestinal lumen and so-called villi, which are surrounded by tissue invaginations called crypts, which house the intestinal stem cell niche (Figure 1B). The epithelium of the small intestine turns over every 5 days in mice and constitutes the most rapidly regenerating tissue of adult mammals. Cell production starts at the crypt base, producing numerous progenies, which move up the crypt-villus axis. Intestinal stem cells are clustered at the bottom of the crypts, and cells moving up the crypt continue proliferating while in parallel becoming committed either to an absorptive (enterocytic) or a secretory fate (mainly goblet cells, Paneth cells, and enteroendocrine cells). Cells stop proliferating and differentiate while approaching the crypt-villus junction (Figure 1B). Upon reaching the villus tip a few day later cells are shed into the lumen of the intestine. Transit amplifying cells emanate from the bottom of the crypts. At the most apical part of the crypt, cells stop to divide and the epithelial sheet undergoes fine polarization and specific organization while progressing on the villus, called terminal differentiation, with the formation of a structural and functional additional feature at the apex, a brush border of microvilli, which increases the plasma membrane surface at the apex and thus enhances cell absorption at the interface of the gut lumen and epithelium (Barker, 2014; Delacour et al., 2016).

The epithelial layer of the GI tract is subjected daily to aggression from external elements present in the diet and the external world, and its erosion must be compensated by secretions from the mucosa to guarantee the integrity of the barrier, the first line of defense against external aggression. It is therefore coated with a lubricating protective barrier, the mucus gel, a viscoelastic gel which provides a physical barrier between the underlying luminal surface of the epithelial layer and microorganisms, the toxins they produce and other potentially harmful substances present in the intestinal lumen. Mucus gel is produced by goblet cells, which are disseminated between enterocytes in the upper two-thirds of the crypts but also along the villi. Reaching 15% of the total population of the duodenal epithelium, the proportion of goblet cells gradually increases to 40% in the distal colon (Wang et al., 2019a). Secretory granules produced by goblet cells contain the mucins, the main structural component of the mucus gel. MUC1, MUC2, MUC3, MUC4, and MUC5AC are the mostly expressed mucins in humans at the GI level, MUC2 being the main component of the intestinal mucus gel (Hollingsworth and Swanson, 2004; Okumura and Takeda, 2018). Mucins are responsible for its viscoelastic and gelling properties and display the common characteristics of being high molecular weight glycoproteins (up to 30,000 kDa) where carbohydrate chains represent up to 80% of the weight of the mucin (Pinho and Reis, 2015). This high glycosylation state gives mucins high density and viscosity properties. In addition to its physical and chemical protective function, the mucus gel is in symbiosis with the endogenous bacterial flora. It offers many benefits to bacteria in the intestinal lumen since mucins can provide a direct source of nutrients for bacterial growth, and the gel structure facilitates the colonization of the intestine by bacteria, which can survive and multiply (Sicard et al., 2017; He et al., 2018).

The GI tract is also a major site of immune surveillance and its lumen is inhabited by a microbial community called the gut microbiome (Figure 1C) (Round and Mazmanian, 2009; Garrett et al., 2010), which plays an important role in normal intestinal function and has been implicated in numerous intestinal diseases including inflammatory bowel disease, gastrointestinal malignancy, and celiac disease (Clemente et al., 2012; Lloyd-Price et al., 2016). Additionally there exist stroma, immune cells (e.g., Peyer’s patches in small intestine, macrophages, and neutrophils), and an endothelium/blood supply (Drake et al., 2010). The immune system monitors for and suppresses pathologic gastrointestinal infections, and at times it acts aberrantly, leading to diseases like inflammatory bowel disease and celiac disease (Figure 1C).

*In vivo* and *in vitro* models of gastrointestinal function are important tools as they allow many experiments that are unfeasible and unethical to do in humans. *In vivo* studies on animal models such as mice or pigs have been extensively used to understand the normal and pathological development of the intestinal organ. However, they have been largely restricted due to ethical and financial problems, weak reproducibility, variability of individuals and the difficulty to isolate the influence of one given factor. Moreover, as the animal physiology differs from human physiology, animal models only reflect a few aspects of human diseases. As another option, *in vitro* 2D cell culture models, such as those using Caco-2, T84 cell or HT29 lines, are frequently used to study the GI tract, including nutrient transport, intestinal absorption, cell differentiation and human diseases including carcinogenesis (Hilgers et al., 1990; Nataro et al., 1996; Delacour et al., 2003). However, the use of established cell lines intrinsically displays several major drawbacks. They only contain the enterocytic cell type and do not allow functional analyses representative of the entire intestinal epithelium. Moreover, established intestinal epithelial cell lines such as Caco-2 cells are of cancer origin and harbor multiple gene mutations, which could incur problems in genome fidelity and personalized medicine approach (Kasendra et al., 2018). For all those reasons, development of alternate intestinal *in vitro* models is crucial for the progression of the gastroenterology field of research.

## Intestinal Organoids and Their Limitations

In 2009, Hans Clevers’s work revolutionized the intestinal bioengineering field with the generation of a mouse intestinal organoid *in vitro* model system called “mini-gut” or “enteroid” (Sato et al., 2009), which was then followed by the establishment of a human version either from human adult stem cells (AdSC) or pluripotent stem cells (PSC) (Spence et al., 2011). Since then, organoids have become a very attractive tool for researchers to study intestinal morphogenesis (Sumigray et al., 2018) and homeostasis maintenance in such a dynamic tissue due to their physiological relevance compared to classical 2D cultures of immortalized cell lines. In fact, classical 2D cultures of established cell lines do not allow adequate studies on epithelial organ morphogenesis. Since *in vivo* epithelial cells organize into 2D monolayers experiencing various out-of-plane curvature and 3D geometries, it is essential to study the intestinal epithelium in a topography that is physiologically relevant. Over a decade ago cystogenesis in 3D Matrigel matrix emerged as a key experimental tool to study epithelial morphogenesis *in vitro* for cell line-based cultures. This system brought remarkable progress in the understanding of the sequential events of epithelial arrangement and lumen generation during the development of spherical structures such as tubules or acini (O’Brien et al., 2002). It has been useful in the understanding of different human pathologies based on spherical or tubular formation defects, such as polycystic kidney disease. Nevertheless, this 3D culture system is not appropriate to study epithelial layer maintenance in non-spherical or non-tubular shaped organs, such as along the architecture of the intestinal mucosa. More importantly, it only gives access to the dynamics of a single differentiated cell type during morphogenetic processes, and does not recapitulate the coordinated evolution of various stem and differentiated cell types that takes place in the native intestinal tissue. Initially generated from isolated crypts, intestinal organoids are three-dimensional *in vitro* systems, which house the intestinal stem cell niche. They are essentially “mini-organs” that retain most physiological conditions such as the spatial organization of cells, cell-cell interactions, and cell-matrix interactions (Yin et al., 2016). Organoid culture allows for monitoring of how intestinal tissues develop and maintain homeostasis through different conditions using live-imaging techniques such as two-photon or spinning disk microscopy.

**FIGURE 2:**
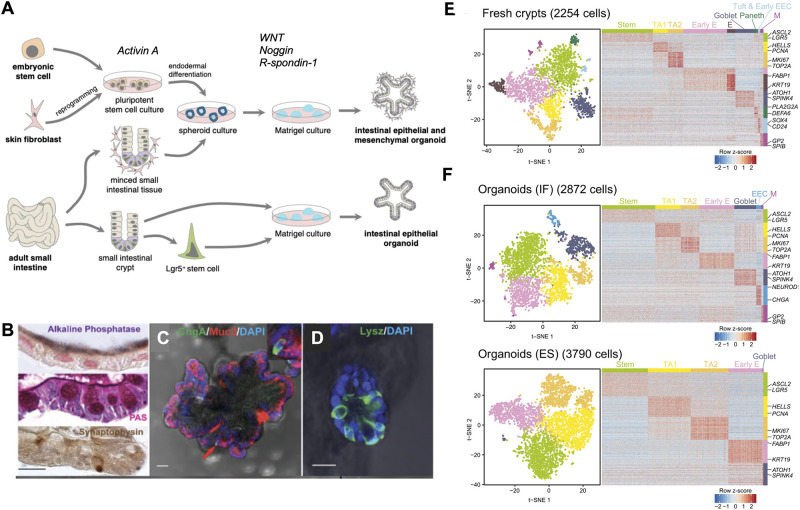
State-of-the-art in intestinal organoids. **(A)** Organoids can be derived from either embryonic stem cells or induced pluripotent stem cells (iPSC) from reprogrammed differentiated cells. iPSCs grown in the presence of Activin A undergo endodermal development and in the presence of niche factors Noggin, EGF, and R-spondin-1, form intestinal organoids containing both epithelial and mesenchymal cell types. Small intestinal tissue samples can be directly cultured as spheroids to produce organoids or LGR5+ stem cells can be isolated from these tissues to produce epithelial organoids when grown in the presence of niche factors. **(B)** Alkaline phosphatase staining for mature enterocytes, periodic acid-Schiff (PAS) staining for goblet cells, synaptophysin staining for enteroendocrine cells. **(C)** Human intestinal organoids expressing Mucin 2 (red) a marker of goblet cells, chromogranin A (green) a marker of enteroendocrine cells, **(D)** lysozyme (green) a marker of Paneth cells. **(E)** Single cell RNA sequencing demonstrates cell types in conserved in fresh human ileal crypts and in **(F)** intestinal organoids cultured under two different conditions, standard organoids cultured with two different culturing conditions: traditional culture conditions (bottom panel) compared to a new set of culture conditions (IGF-1 and FGF-2). Scale bar demonstrates statistical association (*z*-score) of specific genes with specific cell types listed.

With the identification of Lgr5-positive-intestinal stem cells (LGR5+ ISC) and the understanding of the signals controlling ISC behavior in the mouse, Sato et al. (2009, 2011b) were successful in developing *in vitro* murine intestinal organoids, representing a powerful breakthrough in the intestinal research field. Intestinal organoids can be derived from either a single LGR5+ stem cell or from transplanted intestinal crypts by embedding them in Matrigel, a 3D substrate that mimics the complex extracellular environment found in many tissues and is composed of ECM components such as laminin, Collagen IV, entactin, and heparin sulfate proteoglycans. The formation and maintenance of intestinal organoids inside the Matrigel is supported by the addition of a medium containing epidermal growth factor (EGF), R-Spondin-1, and Noggin, which are important to stimulate proliferation and maintenance of stem cells while blocking differentiation. A transplanted crypt in these conditions will close after a few hours to form a spheroid, which then starts budding to develop into a mature organoid with distinct crypt-like structures as seen *in vivo* (Figure 2A). These crypt-like budding structures include LGR5+ stem cells and Paneth cells, whereas in the main body of organoids contains differentiated cells forming the villus-like areas (Figure 1). Other cell types including goblet cells, enterocytes, and enteroendocrine cells (Sato et al., 2009; Kretzschmar and Clevers, 2016) can be identified by immunohistochemistry (Figures 2B–D) and more recently by single cell RNA sequencing (Figures 2E,F) (Grün et al., 2015). Moreover, addition of Wnt3A to the combination of growth factors allowed indefinite growth of mouse colon organoids and addition of nicotinamide, along with an inhibitor of Alk and an inhibitor of p38 is required for long-term culture of human small intestine and colon organoids (Jung et al., 2011; Sato et al., 2011a). Recently, a p38 inhibitor was found to impair proper cellular differentiation in human gut organoids. An effort has been made to identify IGF-1 and FGF-2 as growth factors that can improve human intestinal organoid plating, recovery, self-renewal and differentiation capacity (Fujii et al., 2018).

Alternatively, it is also possible to develop human intestinal organoids using induced pluripotent stem cells (iPSCs) (Spence et al., 2011). Pioneered by S. Yamanaka in 2006, the introduction of defined transcription factors allow the reprogramming of differentiated adult cells from any tissue such as skin or blood pluripotent cells to immature pluripotent cells that have regained the capacity to differentiate into any type of cell in the body (Takahashi and Yamanaka, 2006; Takahashi et al., 2007). In the context of personalized medicine, iPSCs are very useful for constituting patient biobanks and specific therapeutic strategies without any rejection due to immune response (Takahashi and Yamanaka, 2013; Scudellari, 2016). For intestinal engineering, primary cells can be differentiated into endodermal cells in 2D cultures. Then the formation of a gut tube is provoked thanks to Wnt3 or FGF treatment, and gut tubes are transposed in 3D ECM matrix with culture medium complemented with notable EGF, Noggin and R-Spondin-1 factors. In comparison to the classical mini-guts, this alternate organoid culture system develops crypts but also villus-like structures and presents the advantage of an epithelial-mesenchyme co-culture (Wells and Spence, 2014). This human PSC-derived gut organoid often showed immature fetal organoid features that require further maturation *in vitro*. A cytokine-based maturation protocol has been introduced as a step for full maturation of human PSC-derived gut organoids (Jung et al., 2018).

Intestinal organoids have been shown to recapitulate normal intestinal physiology including ion, water, and nutrient absorption/secretion and have been used to model pathophysiologic processes, including intestinal infection and cancer. Importantly, organoids created from primary patient samples can be frozen and banked. These cryopreserved samples can be repeatedly thawed and grown, providing as sample source of human materials. Thus, organoids are a complementary model to cell lines and xenograft models, and very useful in the case of rare patient samples or personalized medicine (Van De Wetering et al., 2015). Because organoids are readily established from individual patient samples, they can be used for personalized medicine studies such as studying particular human mutations or individualized responses to drug treatments (Van De Wetering et al., 2015; Fatehullah et al., 2016; Yin et al., 2016). The versatility of intestinal organoids further allows the application of various biological methods of genetic modification to be performed on intestinal organoids in both mouse and human tissue using different tools such as siRNA, CRISPR/Cas9 editing, lentiviral infection, and inducible systems that incorporate both lentiviral infection and the efficiency of the CRISPR/Cas9 tool, to produce normal and pathological phenotypes with fluorescent markers (Koo et al., 2012; Schwank et al., 2013a, b; De Van Lidth Jeude et al., 2015; Drost et al., 2015; Fujii et al., 2015; Andersson-Rolf et al., 2016, 2017; Broutier et al., 2016; Driehuis and Clevers, 2017). These genetically modified organoids are used in fundamental biology to understand the role of certain proteins in maintaining the proper function and performance of the intestinal tissue. They are also widely used to model diseases such as the rare enteropathy microvillous-inclusion disease (MVID) (Mosa et al., 2018) or colorectal cancer (Drost et al., 2015; Matano et al., 2015). For proper gene-editing of intestinal organoids, researchers have described several protocols with some variations (Miyoshi and Stappenbeck, 2013; Andersson-Rolf et al., 2014; Fujii et al., 2015; Merenda et al., 2017; Fujii et al., 2018).

There are many advantages to organoids as stated above but still many limitations exist, such as (i) ethical aspects of the use of live human derivatives, (ii) the lack of repeatability and quality control of the variability of individual samples collected or used, (iii) the difficulty of isolating the influence of a particular factor in a complex environment. In addition, these culture systems do not consider the fact that cells have to integrate numerous factors of the microenvironment: (i) geometrical: topographical variations of the substrate, (ii) mechanical: substrate rigidity, and (iii) chemical: extracellular matrix proteins, morphogens, molecule diffusion. Importantly, while being appropriate for crypt morphogenesis and dynamics studies, AdSC-derived human intestinal organoids do not form villi *per se* in culture. PSC-derived human intestinal organoids do form villus-like structures but only contain a restricted differentiated compartment, and they have been recently reported to be immature and to actually be much closer to fetal villi. These two culture systems then cannot be used for correct villus epithelium organization analyses. In addition, they have very irregular shapes that can greatly vary from one organoid to another, which can be attributed to the lack of the underlying mesenchymal cells and basement membrane that is replaced with a soft Matrigel *in vitro*. Moreover, they only represent the epithelial layer of the GI tract and they lack other important constituents including immune, stromal, muscular, endothelial/vascular, and microbiome components, which are important for normal intestinal function and which have known roles in various diseases. Organoid models also do not incorporate mechanical motion (peristalsis) and fluid flows that are a part of the normal intestinal function. In addition, they form a closed lumen and the apical aspect of the lumen faces the interior of the organoid. Thus it is difficult to access the luminal component of the cells to study the mucus layer or to evaluate the effect of drugs, toxins, microbes, and other stimuli in these systems (Bein et al., 2018). In this context, microinjection techniques have been used with limited success since the procedure provokes damages to the organoid structure (Bartfeld et al., 2015; Forbester et al., 2015; Wilson et al., 2015; Heo et al., 2018; Williamson et al., 2018).

In summary, though holding great advantages compared to classical 2D cultures, intestinal organoids on their own cannot recapitulate every architectural and physiological aspects of the intestinal tissue. In addition, the need of new intestinal culture systems where one could precisely control as much as possible various physical and chemical parameters has emerged. Thus, the current trend in the field is to use the unique properties of the organoids and to adapt/combine them to material engineering techniques. Development of *in vitro* models of the intestine that recapitulates the structural, absorptive, mechanical, microbial, physiologic, and pathophysiological properties of the human gut could accelerate pharmaceutical development and potentially replace animal testing. This has been the driving force behind biomimetic intestinal engineering and microfluidic “gut on a chip” model development.

## Importance of Biophysical and Biochemical Cues in Designing *In Vitro* Intestinal Models

A major challenge in the field remains to adapt synthetic surfaces to organoid primary cultures. Developed over the last decade, microfabrication techniques allowed the development of synthetic substrates with controlled chemistry and defined geometries at the micron scale, that can be used in cell culture systems (Le Digabel et al., 2010). Such approaches are now used to mimic biochemical and biophysical cellular environments and investigate intestinal epithelial morphogenesis *in vitro* in more physiological conditions. Widely used for culturing established cell lines, they offer the advantage of effectively breaking down cell behaviors in relation to specific and known conditions such as concentrations of adhesion molecules, tissue stiffness or geometric dimensions. However, adapting organoid primary cultures on such microfabricated surfaces is a way more difficult than culturing cancer cell lines, and thus requires a strong knowledge of the intestinal tissue to develop appropriate microfabricated surfaces. This challenge is currently taken up by combining biochemistry and biomaterials to capsulize the heterogenous properties of the cellular intestinal environment.

### Mimetism of the Intestinal Tissue Biochemistry

A difficult task in intestinal tissue engineering is to imitate the underlying lamina propria or extracellular matrix (ECM) which provides a biochemical support for cells within the intestinal tissue. The ECM is composed of the basement membrane, a very dense matrix mainly composed of collagen type IV, laminin and fibronectin, and the interstitial matrix, which constitutes a loose and porous fibrous scaffold constituted of collagens, elastin and fibronectin (Kular et al., 2014; Chen and Liu, 2016). The basement membrane exhibits direct interactions with cellular ECM receptors such as integrins in epithelial cells, influencing cell adhesion, cell growth, migration, gene expression, morphology and differentiation (Rozario and DeSimone, 2010). Indeed, abolishing the contacts between intestinal epithelium with the ECM often impinges on tissue integrity and homeostasis. For instance, the deletion of the integrin alpha-6, an ECM receptor, provokes defects in the epithelial barrier, as well as prolapse formation and colitis-associated adenocarcinoma development in mice (De Arcangelis et al., 2017). Moreover, conditional depletion of laminin gamma-1 in mouse leads to crypt hyperplasia and epithelium detachment along the villus (Fields et al., 2019).

It is important to mention that the ECM composition and architecture differ according to the tissue, and, within the intestinal tissue, additional spatio-temporal heterogeneities occur (Kedinger et al., 2000). Each intestinal segment exhibits a distinct ECM component mixture. For instance, an increasing gradient of collagen-VII has been reported along the antero-posterior axis of the gut (Leivo et al., 1996). Furthermore, differential deposition of ECM component isoforms takes place along the crypt-villus axis: as an example, laminin-1 and -2 being enriched at the level of crypts, whereas laminin-3 and -5 are prominent in villi (Simon-Assmann et al., 1995; Leivo et al., 1996; Simon-Assmann et al., 1998; Teller et al., 2007; Fields et al., 2019). In addition, gene expression of ECM proteins also varies during the gut development (Simon-Assmann et al., 1995). Whereas laminin-1 is downregulated after birth, laminin-3 expression increases at late embryonic and postnatal stages (Teller et al., 2007). It is worth mentioning that ECM composition is frequently altered in intestinal diseases. It is well described that abnormal ECM remodeling contributes to the progression of the inflammatory bowel disease (IBD) and Crohn’s disease (Petrey and De La Motte, 2017). In the case of the congenital tufting enteropathy (CTE), a rare intestinal disease, patient duodenal biopsies display defects of laminin deposition in the basement membrane, which, at least in part, participates to the formation of characteristic epithelial tissue lesions in villi (Goulet et al., 1995; Righini-Grunder et al., 2017). Moreover, the composition, density and integrity of ECM components evolve concomitantly with tumor development and dynamics (Frantz et al., 2010). As an example, increase of collagen-X and alpha-3 chain of collagen-VI takes place in the development of colorectal cancers, making these ECM components potential diagnostic markers (Solé et al., 2014; Qiao et al., 2015). Thus, the choice of the appropriate ECM composition remains a crucial point in the biomimetic approach.

To recapitulate the intestinal native ECM, two approaches can be followed: either using natural ECM matrices or to produce synthetic ones. Natural ECM matrices can be produced upon cell removal from the native intestinal tissue. In fact, decellularized small intestinal submucosa samples from rat or pig has been used in the context of tissue repair or regeneration (Hodde, 2002; Andrée et al., 2013). This source of natural ECM scaffold has been tested for intestinal biomimetic cultures for *in vitro* fundamental research. Protocols have been adapted to generate from animal models or human samples decellularized intestinal tissue that keep crypt-villus structures (Totonelli et al., 2012; Giuffrida et al., 2019), and combination of such natural ECM scaffolds with intestinal organoid cultures allows the generation of a differentiated intestinal model *in vitro* (Finkbeiner et al., 2015).

In order to get closer to the tissue environment, natural biomaterials are used in microfabrication, though they are costly and control of their physical properties cannot be performed. For instance, collagen-based scaffolds give the possibility of more physiological culture systems since it is an intrinsic extracellular matrix (ECM) component and the 3D architecture of the collagen meshwork is close to the *in vivo* context (Millet et al., 2019). However, more complex ECM-based hydrogels can be used. In 1977, R. Orkin pioneered the field with the production of a basement membrane gel from Engelbreth-Holm-Swarm (EHS) chondrosarcoma mouse tumors (Orkin et al., 1977; Kleinman and Martin, 2005). Now known as Matrigel, this hydrogel is constituted of a cocktail of laminin, collagen IV, entactin, heparan sulfate proteoglycans and growth factors such as TGFbeta and FGF. More recently, Giobbe et al. (2019) proposed the preparation of ECM-derived hydrogels from decellularized intestinal submucosa which allows organoid cultures.

Another approach consists in the use of synthetic hydrogel scaffolds Generated as physical support structures, they can be modified by modulating surface biochemistry with addition of ECM components and will mimic, at least to some extent, the biological function of the ECM (Kular et al., 2014). Among the synthetic biomaterials, PEG hydrogels have been extensively used as scaffolds in tissue engineering. PEG is a non-adhesive material, so it constitutes an excellent base for bioactive modifications and selective incorporation of identified bio-functional oligopeptide sequences corresponding to proteins of the ECM whose concentration and spatial distribution can be easily modulated to provide fundamental insight of signaling events involved in specific cell-matrix interactions and repercussion on epithelial organization. Thus, the interaction between cell surface receptors and specific ligands of the ECM are replaced through the chemical attachment of peptides to the hydrogel scaffold. Synthetic peptides are able to bind to cell surface receptors and mediate cell adhesion with high affinity and specificity similar to that observed with intact proteins. Peptides are generally preferable to intact proteins as they are not subject to denaturation and may be less susceptible to proteolysis. The most extensively used peptide is the sequence Arg-Gly-Asp (RGD), which is found in many cell adhesion proteins and binds to integrin receptors (Rozario and DeSimone, 2010; Leijten et al., 2017).

Furthermore, the ECM network stores and sequesters active molecules essential for tissue development and homeostasis (Frantz et al., 2010; Rozario and DeSimone, 2010). A growing body of literature has demonstrated that the spatial patterning of cellular behaviors, such as proliferation and differentiation, is important during tissue development. Control of intestinal cell fate and differentiation have been long shown to be triggered by downstream signaling pathways and morphogens in the crypt-villus axis (Sancho et al., 2004; Nelson et al., 2005; Crosnier et al., 2006). Morphogens are long-range signaling molecules such as growth factors and cytokines that can pattern developing tissues by inducing distinct cellular responses in a concentration-dependent manner. Their graded activity within tissues exposes cells to different signal levels and leads to region-specific transcriptional responses and cell fates. Responding cells often transduce morphogen levels in a linear fashion, which results in the graded activation of transcriptional effectors. The spatial distribution of Wingless/Int (Wnt), Hedgehog (Hh) and bone morphogenetic protein (BMP) has been longstanding reported to direct the organized formation of the intestinal unit (Sancho et al., 2004; Crosnier et al., 2006; Williamson et al., 2018). In tissue engineering, studies report the immobilization of growth factors in hydrogel biomaterials include EGF, bFGF, VEGF, NGF, TGF-beta, either by grafting on PEG framework or by alginate encapsulation and incorporation in the hydrogel scaffolds (Rozario and DeSimone, 2010; Leijten et al., 2017). More recently, Wang et al. (2017b) mimic the effect of the morphogen gradient taking place in the intestinal unit by culturing organoid-based biomimetic 3D substrates on Transwell filters and adding distinct culture media (either enriched in Wnt3a, R-Spondin and Noggin to maintain the stem cell niche, or in DAPT to stimulate epithelial differentiation) in the lower or upper insert compartments, respectively.

### Mimetism of the Intestinal Tissue Stiffness

Besides chemistry, rigidity is another essential property of the ECM microenvironment controlling cell behavior. Cells interact with the environment through cell-substrate adhesions and sense the mechanical status of the ECM. Through the mechanotransduction process, cells can thus adapt in response to the physical properties of the tissue and modulate its organization and homeostasis (Iskratsch et al., 2014). For example, matrix rigidity regulates cell differentiation program. Soft matrices favor the differentiation of mesenchymal cells toward the neurogenic pathway, whereas stiff matrices orient toward the osteogenic pathway (Engler et al., 2006). According to the tissue, stiffness varies in a range from 100 Pa (brain) up to 1 GPa (bone). Recent analyses have determined the stiffnesses of the global human ileum and colon at 0.6 and 1 kPa, respectively (Stewart et al., 2018). However, precise stiffness measurements along the crypt-villus axis, the functional unit of the intestinal epithelium, remain to be performed.

In fact, the intestinal ECM should not be seen as a frozen structure, but rather as a dynamic network. Its stiffness is modulated by its composition and fiber density, the activation of myofibroblasts and their ability to contract ECM fibers, as well as the pathological state of the tissue (Tomasek et al., 2002). In inflammatory bowel disease (IBD) or Crohn’s disease, the increase of ECM density provokes intestinal fibrosis and subsequently exacerbated ECM stiffness (Latella and Rieder, 2017; Stewart et al., 2018). In addition, during tumor development, the modification of the ECM participates in tumor growth, cancer stem cell modulation, propagation and extravasation (Kaushik et al., 2019). This process is enhanced by the additional enzymatic ECM remodeling by matrix metalloproteinases (MMPs). For instance, increase of MMP-9 expression and activity takes place in colorectal cancers, where it notably cleaves collagen-I fibers of the ECM (Biasi et al., 2012). In classical microfabrication approaches, the polydimethyl siloxane (PDMS) has been extensively used since it is cheap, easy to use and biocompatible. However, the PDMS material exhibits a stiffness (2 MPa) close to the bone tissue or glass material, making this material a poor candidate for the mimicry of the intestinal tissue stiffness (Barnes et al., 2017). Material replacement with hydrogels has made possible to tune culture device rigidity. For instance, it has been demonstrated that culture of Caco2-cells on PEG replicates improves their columnar organization and differentiation (Sung et al., 2011; Creff et al., 2019). To mimic *in vivo* ECM remodeling, Gjorevski et al. (2016); Blondel and Lutolf (2019), and Brassard and Lutolf (2019) pioneered the field by fine-tuning a enzymatically controlled modulation of PEG hydrogel rigidity. By culturing intestinal organoids in such dynamic matrices, they could show that a soft matrix favors intestinal differentiation. In sharp contrast, a stiff matrix promotes intestinal stem cells proliferation and organoid expansion. Thereby demonstrating that mechanical state of the ECM directly impinges on the intestinal tissue behavior.

### Mimetism of Intestinal Tissue Geometry

In recent years, accumulating experimental evidence have led to recognition that topological tissue properties have the power of directing a variety of cell functions including cell migration, proliferation and differentiation (Nelson et al., 2006; Baptista et al., 2019; Hannezo and Heisenberg, 2019). However, to date few studies concern the intestinal epithelium. Wang et al. (2017b) have shown that when grown on 3D culture devices recapitulating the crypt-villus axis, intestinal stem cells preferentially colonize crypt-like shapes when placed under morphogen gradient. However, recent studies showed that organoid suspensions self-organize and spontaneously generate crypt-like domains in 2D cultures, without any geometrical clues (Thorne et al., 2018; Altay et al., 2019). Therefore, demonstration of the importance of crypt-villus geometry in intestinal tissue development and homeostasis remains to be provided.

Nevertheless, mimetism of the intestinal tissue geometry represents an active field of bioengineering research. Natural ECM-based 3D scaffolds can be directly produced from decellularized porcine intestinal tissue preparation where crypt-villus structures can be kept with adapted protocols. This system is suitable for further intestinal organoid suspension cultures (Maghsoudlou et al., 2013; Finkbeiner et al., 2015). Although this approach has been used in the clinic for intestinal tissue repair (Andrée et al., 2013; Hoeppner, 2013), decellularized intestinal matrix may constitute a poor mechanical support for long-term cultures. In addition, its intrinsic heterogeneity weakens the expected reproducibility properties in bio-engineering experiments. In microfabrication approaches, topographical features that mimic tissue architecture are usually made by photolithography and soft lithography (Le Digabel et al., 2010; Qin et al., 2010; Xi et al., 2019). Shapes are usually obtained through the generation of wafers by photolithography, i.e., by polymerization of a photoresist or photosensitive material exposed to UV light through a patterned mask. Pattern refinement is obtained using deep reactive ion etching (DRIE) and tapered using the surface technology system inductive coupled plasma (STS-ICP) silicon etcher and oxide deposition (Tezcan et al., 2006; Dixit et al., 2012). Recently, spatial resolution of culture devices has been improved with the laser writing technique of 2-photon lithography with which 3D microstructures below 100 nm can be generated (Accardo et al., 2017; Lemma et al., 2019). Direct molding of 3D scaffolds can be performed by soft lithography using elastomers such as the PDMS. Alternatively, PDMS scaffolds can be used as molds for further 3D hydrogel scaffolds production. PDMS has many favorable properties for development of tissue/organ chips, including its optical clarity, and permeability. However, it is well-known to absorb small hydrophobic drugs, which limits its use in drug discovery and development (Berthier et al., 2012). A microfluidic cell culture system was recently developed from a tetrafluoroethylene-propylene (FEPM) elastomer, which was shown not to absorb several hydrophobic compounds, while maintaining other favorable properties of PDMS, thus making it a potentially useful platform for drug discovery and development in the future (Sano et al., 2019). Moreover, PEG is a non-adhesive polymer and is highly resistant to protein adsorption and cell adhesion. PEG hydrogels are biocompatible and hydrophilic, and they have been extensively used as scaffolds in tissue engineering. Crosslinked PEG hydrogel networks swell extensively in aqueous environments providing a 3D highly swollen network with viscoelastic properties similar to soft tissues enabling diffusive transport and interstitial flow characteristics (Lutolf and Hubbell, 2005). In addition, natural compounds such as collagen nicely mimic the chemistry and the 3D organization of ECM layer, and collagen-based 3D replicates have been used to imitate the 3D architecture of the intestinal tissue. However, low-concentration collagen gel is difficult to mold in microfabrication, and increasing collagen concentration subsequently modifies stiffness and porosity properties of the mimetic matrix (Frantz et al., 2010). Such techniques allow to recreate the surface topography of intestinal crypt-villus axis and to obtain well-defined and well-controlled environments for cell culture. We and others performed the first assays on 3D PDMS scaffolds recapitulating the intestinal villus topology and constraints using the Caco2 cell line (Kim et al., 2014; Salomon et al., 2017). Further improvements have been made through the implementation of the crypt geometry and the replacement of cell lines with primary organoid cells (Wang et al., 2017a, b).

In summary, this section recapitulates the various main approaches which have been developed to imitate as much as possible of the intrinsic biochemical and biophysical properties of the intestinal tissue. A further step is to apply and control the distinct elements of the microenvironment on the biomimetic cultures.

## Microfluidic “Organ-On-A-Chip” Models of the Intestine

Organ chips are microfluidic models of biological systems, in which cells are grown in thin chambers using techniques similar to microchip manufacturing. Incorporation of microfluidics allow accurate control of perfusion of these systems. Over the past several years these systems have evolved to incorporate various channel shapes, multiple intestinal cell types, including primary cells, the microbiome, immune and vascular channels and have thus overcome many of the limitations of previous *in vitro* models (i.e., 2D cell culture, organoids, microbiome metagenomics) (Bhatia and Ingber, 2014; Bein et al., 2018).

### Microfluidic Set-Up

Microfluidic devices contain small channels that cells can be grown on. Chips have been developed with multiple channels divided by a thin membrane, such that multiple cell layers can be seeded in different channels. They are thin enough to perform high resolution imaging and simultaneously provide enough cells to do quantitative biological assays. A polymer called polydimethylsiloxane (PDMS) has frequently been used construct gut chips given it is gas-permeable and its clarity permits high-resolution imaging (Huh et al., 2013). Fluids and gasses can be precisely perfused into these systems to mimic fluid flow parameters of the GI system through chambers flanking the microchannels (Huh et al., 2013). Additionally drugs, toxins, nutrients, and growth factors can be precisely delivered to the apical cell surface, a no limitation of spheroid organoid systems (Vickerman et al., 2008).

Initially immortalized cell lines were used to seed intestinal chip models. These models have been used to evaluate drug absorption (Kimura et al., 2015; Pocock et al., 2017), nutrient absorption (Imura et al., 2009), and barrier function (Odijk et al., 2015). To better simulate the intestinal morphology, methods have been developed to form villous shaped channels, with some preliminary suggestion that this better mimics physiologic intestinal cell behavior (Shim et al., 2017; Wang et al., 2017b).

### Intestine Chip Models

Chip based models of the intestine were initially constructed with immortalized intestinal cell lines (e.g., colon cancer cell lines like Caco-2) (Kim et al., 2014; Salomon et al., 2017). However, these cell lines are of GI tumor origin, display a high degree of protein expression dysregulation and thus do not properly represent normal epithelial physiology. In addition, these cell lines only recapitulate the main differentiated cell type of the intestine, the absorptive enterocytes, meaning that notably no stem cell compartment can be studied using these cells. The primary cultures of organoids partly overcome these difficulties. They are developed using the stem cell compartment itself, the intestinal crypts, meaning they contain the intestinal stem cell niche, made of LGR5-positive cells, Paneth and transit-amplifying (TA) cells, and will be able to produce all differentiated cell types present in the intestinal epithelial layer, notably enterocytes, goblet, enteroendocrine, tuft, and Paneth cells. But, as mentioned earlier, their closed lumens do not give easy access to their luminal side for drug, chemical, toxin treatment, and the inability to incorporate other aspects of the physiologic microenvironment (e.g., differential fluid flow and pulsatile flow) as well as co-culture with microbes.

To solve these issues, several authors have enzymatically lysed organoids into single cells and seeded them in monolayers onto 2D substrates made of collagen or Matrigel (Wang et al., 2017a; Thorne et al., 2018). Some went further with the use of Transwell plates coated with collagen or Matrigel matrix where dissociated cells from organoids are seeded (Tong et al., 2018). These monolayers demonstrated apical-basal organization, gave access for chemical or viral treatment of either cell side differentially (Foulke-Abel et al., 2014; Ettayebi et al., 2016). Altogether, organoid monolayers offer the simplicity of 2D culture, which makes it easier for scientists to investigate due to the diverse tools available to study 2D monolayers compared to complex 3D structures. An elegant study from Thorne et al. (2018) recently showed that dissociated organoids do not randomly arrange on 2D substrates. Quite the opposite, primary intestinal cultures are capable of self-organization, *de novo* segregate undifferentiated and differentiated compartments, and even locally form niche-like compartments (Thorne et al., 2018).

Moreover, 2D organoid monolayer cultures have been instrumental in developing biomimetic systems adapted for mucus secretion analyses. Often underestimated in intestinal bioengineering strategies, the mucus gel layer coating the intestinal epithelial layer nonetheless provides a physical barrier but also a permissive environment for bacterial growth. Classical *in vitro* culture models that are commonly used to study intestinal physiology such as Caco-2 cell lines are not suitable to study the mucus secretion process in the intestinal epithelial layer, since they do not produce the typical intestinal MUC2 normally secreted by goblet cells. However, since classical organoids have an enclosed lumen, the mucus produced is entrapped and inaccessible for proper physiological study. Wang et al. (2019a) recently reported a method to implement a mucus layer on top of a 2D organoid monolayer model. Briefly, this technique, reminiscent of studies applied to the respiratory epithelium, is based on the fact that classical medium-based culture systems dilute the secreted mucus on top of the epithelial layer. Switching to an air-liquid interface culture principle, together with treatment with an intestinal hormone modulating water secretion on the luminal side, allowed the accumulation of a mucus layer above organoid monolayers. More recently, D Sontheimer-Phelps et al. (2020) developed an organoid-based human colon-on-a-chip microfluidic device with a high number of differentiated MUC2-producing goblet cells, which allows the formation of a mucus bilayer. They show the presence of a protective inner mucus layer that is impenetrable to bacteria, and an outer penetrable layer where commensal microbes can be found, with a thickness similar to *in vivo* (Gustafsson et al., 2012). Their system is able to support the differentiation of large numbers of mucus-producing goblet cells similar to levels *in vivo*, while maintaining proliferative cells to ensure long-term culture. These data are crucial to improve the physiological relevancy of 2D organoid monolayers in the context of bacteria interaction or immune response.

Nevertheless, the 2D organoid monolayer models still lack tissue topographical properties as well as many characteristics employable in chip-based models including growing multiple cell types (mesenchymal, vascular endothelial, immune), incorporation of microbial organisms, and pulsatile flow. Intestinal chip models incorporating primary cells have then been developed. For example, fragmented human enteroids were seeded into one chamber of a two-chamber microchip, with primary vascular endothelial cells from the same biopsy sample seeded into the second channel. With incorporation of pulsatile fluid flow, the authors claim that these cells undergo villous-like differentiation, although this is quite a preliminary suggestion based on morphology under confocal microscopy and there are not good controls (i.e., no comparison to *in vivo* villi or comparison to epithelial cells cultured in traditional cell culture). More detailed physiologic evaluation of these suggested villi is needed to determine whether they are in fact physiologically resemble villi (Kasendra et al., 2018).

### Microfluidic Devices to Mimic Peristalsis and Physicomechanical Cues

**FIGURE 3:**
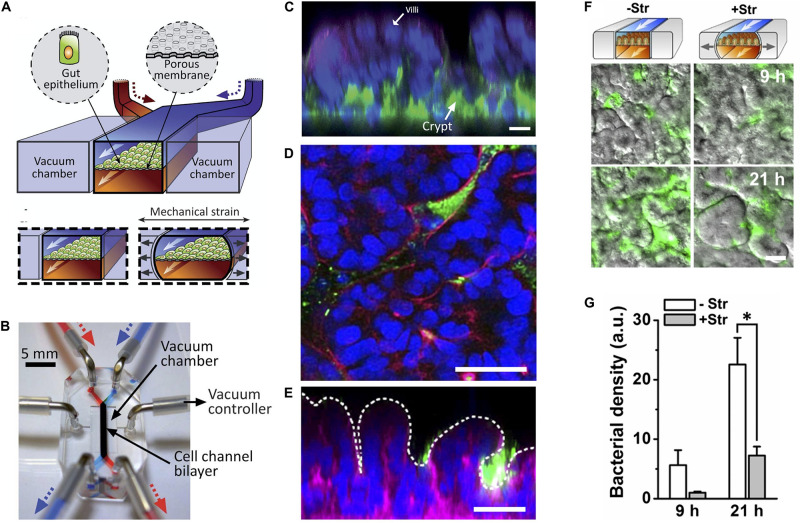
State of the art in gut-on-a-chip platforms. **(A)** Illustration of the cross section of an organ chip with two chambers and flanking vacuum chambers, which are used to perform mechanical deformation. Gut epithelial cells are grown on top of a porous membrane. The upper (epithelial) chamber is perfused by the blue pump and the lower chamber is perfused by the red pump. Two lateral chambers allowed application of vacuum to the side chambers to produce cyclic mechanical strain in the direction of the arrows to mimic peristalsis (Reproduced from Huh et al., 2013 with permission from The Royal Society of Chemistry). **(B)** Photograph of the organ chip constructed from PDMS elastomer with the upper (epithelial) chamber infused by a syringe attached to a pump with a blue dye and the lower chamber being infused with a red dye. The direction of fluid flow is in the direction of the arrows. Cells are seeded in the labeled cell channel. Application of vacuum to produce cyclic mechanical deformation is performed through tubing attached to vacuum chambers flanking the main micro channel using a vacuum controller (Reproduced from Huh et al., 2013 with permission from The Royal Society of Chemistry). **(C)** Caco-2 cells grown under cyclic mechanical strain formed of three-dimensional “villus-like” structures and crypts. Immunofluorescence staining with DAPI nuclear staining (blue), f-actin (green), and mucin staining (magenta). (Reproduced from Huh et al., 2013 with permission from The Royal Society of Chemistry). **(D)** Horizontal and **(E)** vertical cross-sections of green fluorescent protein (GFP) expressing *E. coli* (green) co-cultured with Caco-2 cells on-a-chip (DAPI nuclear stain in blue; F-acin stain in magenta). Dotted line represents the villous surface of these intestinal cells. **(F)** Cessation of mechanical strain (deformation) leads to bacterial overgrowth as seen in Caco-2 cells grown with GFP expressing *E. coli*. **(G)** Quantification of bacterial overgrowth. + Str = cells and bacteria grown in the presence of mechanical strain. – Str = without mechanical strain. (Reproduced from Kim et al., 2016 with permission from The National Academy of Science). *Represents statistical significance as determined by *p*-value < 0.05.

Application of pneumatic cyclic vacuum suction to the side chambers of microfluidic devices leads to pulsatile flow and mechanical deformations, which have been used to mimic peristaltic movements of the GI tract (Figures 3A,B). Evidence suggests that application of mechanical deformation in Caco-2 cells, which normally grow as an epithelial monolayer in 2D culture may undergo three-dimensional villus morphogenesis (Figure 3C) with differentiation into the four intestinal cell subtypes. Morphologically there appear to be villus-like tissue patterning but more rigorous testing is needed to determine how similar these villus-like structures are to actual villi. There is also suggestion of migration of proliferative cells from the basal crypt to the villus tip, formation of the apical brush border, increased cytochrome P450 activity, and enhanced mucus production relative to static cultures which mechanical deformation is applied (Kim et al., 2012; Huh et al., 2013) although we feel the criteria for villus formation needs to be carefully developed and the tissues grown in these models needs more analysis.

It was further found that in Caco-2 cells and intestinal organoid-derived primary epithelial cells, that specific cessation of basal flow, while maintaining luminal flow, halted intestinal morphogenesis and villi formation. By manipulating physical and biochemical cues in the gut chip model, it was identified that the Wnt antagonist DKK-1 was secreted in a polarized basolateral direction and that its removal by fluid flow in the basolateral microchannel is a critical factor that directly triggers intestinal 3D morphogenesis in this model using Caco-2 as well as the primary organoid-derived epithelial cells (Shin et al., 2019). Evaluation of differential stimuli on the luminal and basal sides of the cells was capable on organ chips but not in conventional cell culture, making organ chips a powerful tool for understanding morphogenesis and development. This was further confirmed in a paper by Sunuwar and colleagues which used human jejunal enteroids to show that luminal and basolateral flow produce a model of continual differentiation and NaCl absorption that mimics normal intestine that will be useful in modeling normal intestinal physiology (Sunuwar et al., 2020).

Application of pulsatile flow and mechanical deformation has allowed co-culture of microbes in direct contact with epithelial cells (instead of with a thin membrane) and for the ability to maintain systems for weeks, longer than any static cell culture systems (Kim et al., 2012; Huh et al., 2013). In fact, it was shown that specific cessation of pulsatile flow (stopping the peristalsis-like motion) while maintaining continuous flow, lead to bacterial overgrowth, demonstrating that the lack of pulsatility, not the lack of fluid flow, is the likely mechanism of bacterial overgrowth. This may have implications for the disease small intestinal bowel overgrowth (SIBO), which occurs in the setting of ileus and may suggest that the mechanism of overgrowth has more to do with decreased peristalsis rather than lack of fluid flow in the intestine and that restoring motility may help treat this disease (Kim et al., 2016).

### Incorporation of the Microbiome Into Gut Chip Models

Gut microbiome study has been limited to genomic and metagenomic analysis given difficulty in culturing the largely anaerobic bacterial population. Conventional 2D models are unable to support co-culture of microbiome with cells for extended periods of time limiting analyses because the aerobic bacteria rapidly overgrow and contaminate the cultures in a day. Additionally, obligate anaerobic bacteria are unable to be effectively cultured.

Kim et al. (2012) developed the first microfluidics-based system to co-culture human and microbial organisms using Caco-2 cells and *Lactobacillus rhamnosus* GG for greater than 1 week. Shin and Kim (2018) developed a more sophisticated model called HuMiX (human–microbial crosstalk), which again used co-culture of Caco-2 cells with *L. rhamnosus GG* or *Bacteroides caccae*, this time under anaerobic conditions. Their model demonstrated transcriptional, metabolic and immunologic responses that differed between the two different microbial species (Shah et al., 2016). Limitations of this model include physical separation of intestinal and microbial cells by a thin membrane and lack of pulsatile flow (Kim and Ingber, 2013).

A subsequent model allowed for direct contact between epithelial cells and microbes (Figures 3D,E). This was shown to be possible through the integration of pulsatile flow and mechanical deformation, which produced peristalsis-like waves of deformation (Figures 3F,G) (Kim et al., 2012, 2016) (see section on intestine chip models of peristalsis). They found that co-culture of *L. rhamnosus GG* with intestinal epithelial cells improved intestinal barrier function and also demonstrated that pathogenic bacteria such as enteroinvasive *Escherichia coli* can be integrated into this model (Kim et al., 2016).

One major hurdle to studying the interactions of the comprehensive gut microbiome and intestinal epithelia is that hundreds of bacteria species are obligate anaerobes that will not grow under aerobic conditions (>0.5% O_2_) required to grow intestinal cells (Flint et al., 2012). A recent gut-on-a-chip model overcame this problem by incorporating physiologic oxygen gradients into a gut chip which included primary human ileum epithelium and endothelial layers. Their chip contained two chambers: the epithelial chamber was anaerobic and the vascular (endothelial) chamber grown in aerobic conditions. These chambers were separated by PDMS. Diffusion of oxygen from the aerobic chamber produced an oxygen gradient with oxygen concentration <0.3%. The authors fabricated oxygen sensors with embedded oxygen-quenched fluorescent particles along the epithelial and vascular channels to for real-time non-invasive monitoring of oxygen levels. They grew Caco-2 cells and primary ileal epithelium in this chip. They demonstrated that the obligate anaerobe *Bacteroides fragilis* was able to grow in this system and demonstrated improved growth when anaerobic conditions were applied. They used microbiota originally derived from healthy human stool specimens in order to produce the first *in vitro* microbiome epithelium co-culture. This oxygen gradient allowed for the stable co-culture of the microbiome community in the same channel as mucus-producing human villus intestinal epithelium for 5 days and again showed improved growth of obligate anaerobes in the anaerobic system (Jalili-Firoozinezhad et al., 2019).

Incorporation of the gut microbiome on-a-chip opens doors for many questions previously unanswerable such as how microbial composition differs on the cell surface versus lumen, how specific bacteria interact with host cells and tracking of these dynamics over time in response to stimuli. Additionally, immune cells, epithelium from different regions of the intestine, and specific microbial aspirates from those regions can be added to this system to model specific geographic regions along the GI tract (Poceviciute and Ismagilov, 2019).

### Gut Inflammation On-a-Chip

Chip based models have been used to study gut inflammation and inflammatory bowel disease (IBD). Addition of lipopolysaccharide (LPS) endotoxin to a microfluidic model of the epithelial microchannel demonstrates secretion of proinflammatory cytokines interleukin (IL)-1beta, IL6, IL8, and tumor necrosis factor (TNF)-alpha which lead to increased expression of intercellular adhesion molecule (ICAM)-1, villous blunting, and intestinal barrier dysfunction similar to that seen in inflammatory bowel disease (Kim et al., 2016). An intestinal chip model was also used to study radiation induced gut injury (Jalili-Firoozinezhad et al., 2018).

More recently, in order to identify the initiating factors in gut inflammation, a gut chip incorporating the well-established dextran sodium sulfate (DSS)-induced colitis model (Solomon et al., 2010; Chassaing et al., 2014) was used to assess the effects of gut epithelial barrier dysfunction (DSS treatment), microbial pathogens (*E. coli* or its byproduct LPS), immune components [peripheral blood mononuclear cells (PBMC)], probiotics in various combinations. They demonstrated epithelial barrier dysfunction, pathogenic bacteria (*E. coli* or LPS), and immune cells were required to get gut inflammation and that pretreatment with a probiotic (VSL #3) was able to suppress gut inflammation (Shin and Kim, 2018).

Another study using an intestinal chip model demonstrated that LPS disrupted the intestinal barrier and that lactobacillus was able to protect against the invasion across the epithelium of the opportunistic pathogen *Candida albicans* in this model (Maurer et al., 2019). Another model of enterohemorrhagic E. coli (EHEC) infection, a pathogenic bacterium causing diarrheal illness, showed that addition of specific metabolites from the gut microbiome potentiate the pathogen’s infection, providing a potential explanation for why certain organisms (e.g., mice) and certain human subpopulations (e.g., children) may have different susceptibilities to EHEC (Tovaglieri et al., 2019).

Recently, a gut-on-a-chip model was used to study the relationship of the entero-invasive intestinal pathogen *Shigella* and colonic mucosa. *Shigella* is a pathogen bacillary dysentery, a severe diarrheal illness, with a small inoculation, as few as a few hundred bacteria; however, *Shigella* are particularly hard to culture using traditional cell culture methods (Sansonetti et al., 1996). A gut-on-a-chip system with incorporation of peristalsis-like activity (as described above, see section on “Models of Peristalsis in Intestinal Chips Models”) (Kim et al., 2016) was found to increase the capability of this bacterium to invade human colonic epithelium (Grassart et al., 2019). This provides an important animal-free model that more accurately models Shigella infection that traditional methods.

### Models of Metabolism

The intestine is an important contributor to drug absorption and metabolism that is understudied. First pass metabolism — drug metabolism that occurs before systemic circulation of drugs is a major barrier to drug bioavailability. Traditionally thought to occur in the liver, it has been more recently identified to occur in the intestine as well (Thummel, 2007; Thelen and Dressman, 2009). Animal models are not great for predicting bioavailability for numerous reasons (Martignoni et al., 2006; Paine et al., 2006; Komura and Iwaki, 2011). Human *in vitro* models of intestinal drug absorption have generally used Caco-2 cell lines in cell culture or Transwell plates. Issues with these systems include lack of three-dimensional cellular architecture, lack of other cell populations present in the gut, altered expression profiles of drug transporters and drug metabolizing enzymes, especially CYP450s, and aberrant CYP450 induction response, making use of these models challenging for predicting clinical human responses (Sun et al., 2008). Human intestinal microsomes are more commonly used for gut metabolism rather than Caco-2 cells since they lack CYP450 activity (Hatley et al., 2017).

Several intestinal chip models of drug metabolism were recently developed as possible low-cost, *in vitro* platforms for monitoring drug metabolism. The first used Caco-2 cells to evaluate bioavailability of two drugs (Guo et al., 2018). A second drug metabolism model was adapted from the primary cell human duodenum-on-a-chip model (Kasendra et al., 2018). This model demonstrated polarized cell architecture, intestinal barrier function, presence of specialized cell subpopulations, and expression, localization, and function of major intestinal drug transporters. Notably, when compared to Caco-2 cells, this model displayed improved CYP3A4 expression and induction capability (Kasendra et al., 2019). Lastly, a “functional coupling” organ chip model incorporating intestine, liver, kidney, blood–brain barrier, and skeletal muscle was used to screen drugs to identifying multi-organ toxicity and absorption, distribution, metabolism and excretion. In this publication, each microphysiologic system was run separately in labs across the US and the samples from each chip were shipped to each lab. This operation is not practical for drug screening and questionable to be useful for this purpose, which led to the development of multi-organ chips (Vernetti et al., 2017).

### Gut Chips of the Different Regions of the Intestine

The gastrointestinal system from the mouth to anus is subdivided based on anatomic and physiologic function into the esophagus, stomach, small intestine (consisting of duodenum, jejunum, and ileum), large intestine, rectum, and anus. Organ chip models of the various gut regions have been constructed. A duodenum on a chip constructed using biopsy samples and was shown to transcriptionally more closely resemble *in vivo* duodenum than organoids (Kasendra et al., 2018). An organ chip derived from human ileum samples was used to understand mucosal microbiome interactions (Jalili-Firoozinezhad et al., 2019). This model is more extensively described in the above section entitled “*Incorporation of the Microbiome Into Gut Chip Models.”* More recently an organ chip constructed from human colonic mucosal tissue was developed to study mucus formation. This model demonstrated a subpopulation of proliferative epithelial cells, goblet cells, and the accumulation of a mucus bilayer with thickness similar to what is seen *in vivo.* In response to prostaglandin E2 the mucus layer underwent volume expansive. This model was responsive to prostaglandin in a Na-K-Cl cotransporter 1 dependent manner (Sontheimer-Phelps et al., 2020). There currently is not an organ chip model of the esophagus, although esophageal organoids have been developed from human pluripotent stem cells. Future development of an esophagus-on-a-chip will be an important direction. Another important future direction will be the further use of current region specific gut chips for investigation of specific physiology and disease processes pertaining to these regions.

### Multi-Organ Chips

Multi-organ chips attempt to model multiple organ systems through the culture of multiple cell types that are connected in a microphysiologic system. In one of the seminal multi-organ chip models, Tsamandouras et al. (2017) demonstrate the quantitative contribution of gut and liver microphysiologic systems to drug absorption and metabolism as individual and interconnected MPSs. A three-compartment microfluidic “digestion-on-a-chip” model system was created to study digestion *in vitro*. This was constructed using three compartments in series to mimic the environments of the mouth, stomach, and small intestine. Each compartment was with specific pH, buffer, and mineral composition as well as saliva, gastric, and duodenal fluid in order to mimic their respective local physiologic environment. Digestion of starch, casein, and milk protein (lactoferrin) were used in the study as model nutrients and enzyme kinetics were monitored in real time across the system. Notably, no gut mucosa was used in these models, which limits its current use for the study of drug metabolism and bioavailability (De Haan et al., 2019).

Another study developed a microfluidic platform connecting the liver, stomach, and intestinal cells in a multi-organ-on-a-chip, with the goal of using this as an *in vitro* drugs screening platform, given that not only the liver, but the intestine and stomach play roles in drug metabolism (Jin et al., 2018).

Recent studies suggest peripheral inflammation and microbial pathogens could be triggers for neurologic diseases like Alzheimer’s (AD) and Parkinson’s disease (PD) (Dominy et al., 2019; Rekdal et al., 2019). Recently the European Research Council (ERC) funded a project called “MINERVA” aimed at evaluating the effect of intestinal microbiota on brain functionality through development of a gut-brain chip model. This model has five miniaturized organs (gut microbiota, gut epithelium, immune system, blood–brain-barrier, and the brain) connected hydraulically through a microfluidic system. The goal of this work is to test the effect of microbiota from AD versus healthy patients in the system (Raimondi et al., 2019).

## Conclusion and Future Perspectives

In this review, we describe the recent efforts to develop micro-physiological models of the GI system. The overarching goal of organ-on-a-chip technology is to emulate *in vivo* system physiology in a highly controllable environment. Despite the significant listed efforts to model the GI tract *in vitro*, there remain significant challenges and room for improvement in model development.

We still can only model a handful of cell types at a time. Being able to interface numerous cell types at once will be a challenge but is important to recapitulate many processes. For example, modeling the microbiome interactions with epithelium is interesting but the human immune system is very important for pathogen response and in the induction of tolerance (Belkaid and Hand, 2014). Some incorporation of the immune system into organ chips is currently possible but it is largely limited to infusion of peripheral blood mononuclear cells and injection of various cytokines. Furthermore, future work needs to identify ways to integrate the ENS, which is recognized as having an increasingly important role in the regulation of stress response, blood flow, secretion, metabolism, and immune regulation (Sharkey et al., 2018).

Organ-on-a-chip technology will be quite powerful for advancing personalized medicine approaches. Organoids can be used to do some personalized studies, but they lack the ability to incorporate multiple tissue types and their luminal surface is on the interior of the organ and so not directly open to drug treatment (as described above); thus, they are limited in their use in personalized medicine approaches. Organ-on-a-chip technology will allow growth of patient-specific samples in the presence of microbiome and immune systems and so will more fully incorporate these factors in.

Organ-on-a-chip technology is creating a new wave of powerful *in vitro* tools that overcome many of the limitations of traditional cell culture and have begun to allow many complex biomedical questions to the asked that were unattainable with previous technology. There remain many avenues to improve this technology to make it more biologically relevant as it becomes an increasingly important tool in biomedical research.

## Author Contributions

SS, JS, B-KK, DD, and D-HK wrote the manuscript. SS constructed the figures. All authors contributed to the article and approved the submitted version.

## Conflict of Interest

The authors declare that the research was conducted in the absence of any commercial or financial relationships that could be construed as a potential conflict of interest.
